# Evaluation of the ovarian reserve function in patients with metabolic syndrome in relation to healthy controls and different age groups

**DOI:** 10.1186/1757-2215-7-63

**Published:** 2014-06-10

**Authors:** Fevzi Balkan, Nurcan Cetin, Celil Alper Usluogullari, Oguz Kaan Unal, Betul Usluogullari

**Affiliations:** 1 Endocrinology and Metabolic Disease, Aksaray State Hospital, Aksaray, Turkey; 2 Radiology Department, Aksaray State Hospital, Aksaray, Turkey; 3 Endocrinology and Metabolic Disease, Dr. Ersin Arslan State Hospital, Gaziantep, Turkey; 4 Endocrinology and Metabolic Disease, Aksaray State Hospital, Aksaray, Turkey; 5 Gynecology and Obstetrics Department, Cengiz Gokcek State Hospital, Gaziantep, Turkey

**Keywords:** Metabolic syndrome, Ovarian reserve, Ovarian volume, Antral follicle, Obesity, Gonadotropins

## Abstract

**Objective:**

To evaluate the ovarian reserve function in female patients with metabolic syndrome (MetS).

**Methods:**

This study evaluated 136 subjects, 67 with MetS and 69 controls. Subjects were divided into three age groups. Group I included 49 subjects aged 20–29 years, 22 with MetS and 27 controls; group II included 45 subjects aged 30–39 years, 22 with MetS and 23 controls; and group III included 42 subjects aged 40–49 years, 23 with MetS and 19 controls. Demographic characteristics, anthropometrics, blood biochemistry, and gonadotrophic hormones were compared as total ovarian volume and antral follicle count on ovarian transvaginal ultrasonography.

**Results:**

Serum levels of FSH, LH, E2 and progesterone were similar in the MetS and control groups, while testosterone levels were significantly higher in MetS patients than controls, both in the overall population (p = 0.024) and in those aged 20–29 years (p = 0.018). Total ovarian volume was significantly lower in MetS patients than controls, in both the overall population (p = 0.003) and those aged 20–29 years (p = 0.018), while antral follicle counts were similar. Ovarian volume correlated positively with antral follicle count (AFC) (r = 0.37; p < 0.001) and negatively with age (r = 0.34; p < 0.001) and FSH concentration (r = 0.21; p = 0.013). AFC was negatively correlated with age (r = 0.36; p < 0.001).

**Conclusion:**

Ovarian reserve function is significantly lower in MetS patients than in healthy control subjects, particularly in women aged 20–29 years.

## Introduction

Female fertility, by spontaneous conception or assisted reproductive techniques, decreases significantly with age, a reduction that may be due to the reduction in the number of primordial follicles over time [1-3]. Ovarian reserve is a measure of the reproductive potential of a woman in relation to primordial follicle count and oocyte quality. Follicle response to stimulation and fertility promoting medications is reduced in parallel to the reduction in ovarian reserve. Determinants of ovarian reserve include basal FSH and inhibin B levels (4), estradiol and LH concentrations, LH/FSH ratio, response to stimulation with gonadotropin releasing hormone (GnRH), ovarian volume, total antral follicle count (AFC) and ovarian stromal blood flow [4,5]. A meta-analysis found that AFC was a better predictor of ovarian response than basal FSH level [6], and several studies have shown that AFC, as determined by high-resolution transvaginal ultrasonography, is a significant predictor of ovarian response [4-7].

Insulin resistance plays a central role in metabolic syndrome (MetS) [8,9]. Obesity has been associated with multiple adverse reproductive outcomes in both males and females [10], although the exact mechanisms are largely unknown. The complexity of the human reproductive system makes identification of the mechanisms linking obesity and adverse reproductive functioning challenging [11].

Despite MetS having a negative impact on fertility in women of reproductive age, cross-sectional evidence suggests that increased ovarian reserve is associated with a healthier cardiometabolic risk factor profile [12]. To our knowledge, however, the effect of MetS on ovarian functions has not been previously investigated. Therefore the present study was designed to evaluate ovarian reserve function in female patients with MetS in Turkey in relation to healthy controls and among different age groups.

## Methods

### Study population

The study included 136 subjects, comprising 67 patients with MetS and 69 healthy controls, who were evaluated at the Department of Endocrinology and Metabolic Diseases, Aksaray State Hospital, between January and July 2013. Subjects were divided into three groups by age. Group I consisted of 49 subjects aged 20–29 years, including 22 MetS patients and 27 controls; group II consisted of 45 subjects aged 30–39 years, including 22 MetS patients and 23 controls; and group III consisted of 42 subjects aged 40–49 years, including 23 MetS patients and 19 controls. Subjects were included if they had a history of spontaneous pregnancy, regularly menstruated at intervals of 21–35 days, had cycle length variations of less than 4 days and had both ovaries. Subjects were excluded if they had a history of chronic renal or liver failure, autoimmune or connective tissue disease, a known malignancy, a history of smoking, known infertility, gynecological abnormalities such as dysfunctional uterine bleeding or menorrhagia, a history of ovarian surgery, or if they had used a hormonal preparation or dubious herbal product within the 3 months prior to enrollment.

Following a detailed explanation of the objectives and protocol of the study, written informed consent was obtained from each subject. The study was conducted in accordance with the ethical principles stated in the Declaration of Helsinki and was approved by the institutional ethics committee of Aksaray State Hospital.

### Study parameters

Demographic and clinical characteristics were recorded for each subject, including anthropometric factors, such as weight, height, body mass index (BMI), waist circumference, and waist-to-hip ratio (WHR). Blood biochemistry parameters included fasting blood glucose (FBG), insulin, high density lipoprotein-cholesterol (HDL-C) and triglyceride (TG) concentrations and homeostasis model assessment-IR (HOMA-IR). Serum concentrations of gonadotrophic hormones were measured, including follicle stimulating hormone (FSH), luteinizing hormone (LH), estradiol (E2), progesterone, and testosterone. Total ovarian volume (cm^3^) and AFC were determined by ovarian transvaginal ultrasonography.

### Assessment of MetS

A diagnosis of MetS was based on the criteria of the National Cholesterol Education Program (NCEP)-ATP III and included three of the following five factors: (i) abdominal obesity, defined as a waist circumference (WC) > 102 cm in men and > 88 cm in women; (ii) serum TGs ≥ 150 mg/dl; (iii) serum HDL < 40 mg/dl in men and < 50 mg/dl in women; (iv) blood pressure > 130/85 mmHg; and (v) FBG > 110 mg/dl [13].

### Assessment of insulin resistance

The estimate of insulin resistance by HOMA-IR score was calculated with the formula fasting serum insulin (lU/ml) × FBG (mmol/l)/22.5 as described by Matthews and coworkers [14]. The cut-off value was taken as 2.7 for HOMA-IR [15].

### Anthropometric measurements

Height, weight, and WC were measured by a designated physician. WC was measured with a folding tape at the natural waistline (the level of the umbilicus) in a horizontal plane. BMI was calculated by dividing body weight (kg) by the square of height (m), and WHR was calculated by dividing waist by hip circumference.

### Blood biochemistry analysis

Venous blood samples were taken from the antecubital regions of all subjects between 08:00 am and 09:00 am after an overnight fast of 8–12 hours. Serum FPG concentrations were measured by the hexokinase method. Serum FSH, LH, total testosterone, E2, progesterone and insulin were measured with specific electrochemiluminescence immunoassays (Elecsys 2010 Cobas, Roche Diagnostics, Mannheim, Germany). Totalcholesterol, HDL-C and TG concentrations were measured using enzymatic colorimetric assays by spectrophotometry (Abbott Architect C16000).

### Assessment of ovaries by transvaginal ultrasonography

Transvaginal ultrasonographic examinations, using a Toshiba Aplio 500 device fitted with a 6-MHz transvaginal probe, were scheduled for between the second and fourth day of menstruation, the same day the blood samples were obtained. All ultrasonographic examinations were performed by a previously designated and experienced radiologist (N.C.) who was blinded to all patient information. The length, width, and height of each ovary were measured in the sagittal and coronal plains. Ovarian volume was calculated using the ellipsoid formula (length × width × height)/6. The number of antral follicles <10 mm in each ovary was counted. Total ovarian volume (right plus left ovarian volume) and total AFC in the left and right ovaries were calculated.

### Statistical analysis

Statistical analysis was performed using SPSS for Windows 17 software (SPSS Inc., Chicago. IL). Normality of distribution of continuous variables was evaluated using the Shapiro Wilk test. Means were compared using Student’s *t* tests or Mann–Whitney *U* test, as appropriate. Spearman’s rho test was used for correlation analysis of factors in the MetS groups. A p value <0.05 was considered statistically significant.

## Results

### Demographic characteristics and anthropometric and laboratory findings

In the overall population, mean ± SDBMI (36.1 ± 5.7 vs. 27.1 ± 4.8 kg/m2, p < 0.001), WC (106.0 ± 13.1 vs. 83.8 ± 12.3 cm, p < 0.001), and insulin (15.1 ± 5.9 vs. 10.1 ± 5.4 IU/ml, p < 0.001), FBG (94.8 ± 10.3 vs. 91.6 ± 8.5 mg/dl, p = 0.049), and TG (159.6 ± 77.5 vs. 113.0 ± 41.3 mg/dl, p < 0.001) concentrations were significantly higher, while HDL-C concentrations (40.8 ± 10.8 vs. 49.7 ± 10.2 mg/dl, p < 0.001) were significantly lower among patients with MetS than in control subjects (Table 1). When grouped by age (20–29, 30–39, and 40–49 years), mean BMI (p < 0.001 each), WC (p < 0.001 each), HOMA-IR (p = 0.002, p = 0.005 and p = 0.02, respectively) and insulin concentrations (p = 0.002, p = 0.005 and p = 0.01, respectively) were significantly higher in patients with MetS than in control subjects.

**Table 1:** Demographic characteristics, anthropometrics and laboratory findings in study groups

	**Study groups according to age**									**Total (n = 136)**		
	**20–29 years (n = 49)**			**30–39 years (n = 45)**			**40–49 years (n = 42)**					
	**MetS (n = 22)**	**Control (n = 27)**	**p value**	**MetS (n = 22)**	**Control (n = 23)**	**p value**	**MetS (n = 23)**	**Control (n = 19)**	**p value**	**MetS (n = 67)**	**Control (n = 69)**	**p value**
Age (yr)	24.0 ± 5.1	23.9 ± 3.8	0.900	34.1 ± 3.1	33.7 ± 2.9	0.700	42.5 ± 2.9	43.1 ± 2.8	0.48	33.7 ± 8.2	32.5 ± 8.5)	0.376
BMI (kg/m^2^)	34.1 ± 5.6	23.8 ± 4.4	<0.001	35.9 ± 5.8	28.1 ± 3.2	<0.001	38.1 ± 5.2	30.5 ± 4.1	<0.001	36.1 ± 5.7	27.1 ± 4.8	<0.001
WC (cm)	110.8 ± 11.6	83.8 ± 11.0	<0.001	107.2 ± 14.4	86.3 ± 9.7	<0.001	108.9 ± 13.9	91.6 ± 13.1	<0.001	106.0 ± 13.1	83.8 ± 12.3	<0.001
HOMA-IR	3.6 ± 1.7	2.2 ± 1.3	0.002	3.7 ± 1.8	2.2 ± 1.4	0.005	3.4 ± 1.3	2.4 ± 1.3	0.020	3.7 ± 1.6	2.3 ± 1.3	0.268
FBG (mg/dl)	92.7 ± 5.5	88.9 ± 6.8	0.043	97.2 ± 13.5	92.2 ± 9.0	0.150	94.5 ± 10.3	94.5 ± 9.2	0.980	94.8 ± 10.3	91.6 ± 8.5	0.049
TG (mg/dl)	135.2 ± 74.7	104.0 ± 28.8	0.052	169.0 ± 65.3	119.1 ± 32.7	0.002	173.8 ± 87.7	118.3 ± 61.1	0.020	159.6 ± 77.5	113.0 ± 41.3	<0.001
HDL-C (mg/dl)	41.2 ± 12.4	51.3 ± 9.5	0.002	41.9 ± 12.2	48.0 ± 10.2	0.080	39.4 ± 7.1	49.3 ± 11.2	0.001	40.8 ± 10.8	49.7 ± 10.2	<0.001
Insulin (IU/ml)	15.9 ± 7.3	10.1 ± 5.4	0.002	14.8 ± 5.5	9.8 ± 5.5	0.005	14.5 ± 4.5	10.3 ± 5.6	0.010	15.1 ± 5.9	10.1 ± 5.4	<0.001

Data are shown as Mean (SD). BMI: Body mass index; FBG: fasting blood glucose; HDL-C: high density lipoprotein cholesterol; MetS: metabolic syndrome; TG: triglyceride; WC: waist circumference; WHR: waist to hip ratio.

### Gonadal hormone concentrations

Serum concentrations of FSH, LH, E2 and progesterone were similar in overall patients and controls, as well as in each of the age groups (Table 2). Testosterone concentrations, however, were significantly higher in MetS patients than in controls, both in the overall population (0.2(0.1) vs. 0.2(0.1) ng/ml, p = 0.024) and in the 20–29 age group (0.3(0.1) vs. 0.2(0.1) ng/ml, p = 0.018).

**Table 2:** Gonadal hormones and radiological findings in the study groups

	**Study groups according to age**									**Total (n = 136)**		
	**20–29 years (n = 49)**			**30–39 years (n = 45)**			**40–49 years (n = 42)**					
	**MetS (n = 22)**	**Control (n = 27)**	**p value**	**MetS (n = 22)**	**Control (n = 23)**	**p value**	**MetS (n = 23)**	**Control (n = 19)**	**p value**	**MetS (n = 67)**	**Control (n = 69)**	**p value**
**Gonadal hormones**												
FSH (mIU/ml)	7.5 ± 8.5	5.9 ± 1.4	0.31	6.2 ± 2.4	6.3 ± 2.3	0.87	7.9 ± 3.4	8.0 ± 3.5	0.95	7.3 ± 5.4	6.6 ± 2.6	0.381
LH (mIU/ml)	5.7 ± 2.9	5.5 ± 2.1	0.77	5.6 ± 2.7	5.3 ± 2.4	0.72	6.3 ± 3.5	5.6 ± 2.3	0.46	5.92 ± 3.1	5.5 ± 2.2	0.380
FSH/LH	1.5 ± 1.7	1.2 ± 0.5	0.4	1.3 ± 0.6	1.4 ± 0.8	0.69	1.4 ± 0.5	1.5 ± 0.6	0.65	1.41 ± 1.1	1.34 ± 0.7	0.593
E2 (pg/ml)	35.8 ± 16.4	36.1 ± 14.9	0.94	35.3 ± 17.0	38.3 ± 15.0	0.53	41.4 ± 18.6	39.4 ± 13.1	0.7	37.6 ± 17.4	37.8 ± 14.4	0.940
Progesteron (ng/ml)	0.4 ± 0.2	0.8 ± 1.6	0.37	0.5 ± 0.8	0.6 ± 0.8	0.73	0.5 ± 0.8	0.4 ± 0.4	0.5	0.5 ± 0.7	0.6 ± 1.1	0.495
Testosteron (ng/ml)	0.3 ± 0.1	0.2 ± 0.1	0.018	0.2 ± 0.1	0.2 ± 0.1	0.17	0.2 ± 0.1	0.2 ± 0.1	0.57	0.2 ± 0.1	0.2 ± 0.1	0.024
**Radiological findings**												
Total ovarian volume (cm^3^)	7.5 ± 2.8	9.3 ± 2.3	0.018	7.0 ± 2.0	8.5 ± 3.2	0.07	5.9 ± 2.2	6.1 ± 1.4	0.77	6.8 ± 2.4	8.2 ± 2.8	0.003
Total antral follicle count	24.7 ± 17.9	28.5 ± 11.9	0.37	24.1 ± 21.6	23.4 ± 14.1	0.89	12.7 ± 13.0	8.1 ± 5.5	0.152	20.4 ± 18.4	21.2 ± 14.1	0.77

Data are shown as Mean (SD). E2. Estradiol; FSH: Follicle stimulating hormone; LH: Luteinizing hormone; MetS: metabolic syndrome.

### Radiological findings

Total ovarian volume was significantly lower in MetS patients than in controls, both in the overall population (6.8 ± 2.4 vs. 8.2 ± 2.8 ng/ml, p = 0.003) and in the 20–29 age group (7.5 ± 2.8 vs. 9.5 ± 2.3 ng/ml, p = 0.018). AFCs were similar in patient and control groups, both in the overall population and the three age groups. Moreover, AFC did not correlate with age, either in the patient or control groups (p > 0.05 each).

### Correlation of ovarian reserve function with clinical, laboratory and radiological findings

Ovarian volume correlated positively with AFC (r = 0.37; p < 0.001), while correlating negatively with age (r = 0.34; p < 0.001) and FSH concentration (r = 0.21; p = 0.013). AFC was correlated negatively with age (r = 0.36; p < 0.001).

## Discussion

This study, the first to investigate ovarian reserve function in women with MetS, found that ovarian reserve, as determined by ovarian volume, was significantly lower in patients with MetS than in healthy controls, in particular in women aged 20–29 years. Moreover, ovarian volume was found to be positively correlated with AFC, and ovarian volume and AFC were negatively correlated with age.

Ovarian reserve can be determined by measuring AFC and ovarian volume by ultrasonography during the early follicular phase. Although we found that ovarian volume was significantly lower in MetS patients than in controls, AFC tended to be lower and FSH concentrations higher in the former, a finding consistent with the reverse interaction between these two parameters. Nonetheless, due to the positive correlation between ovarian volume with AFC and the negative correlations of both with age in our study population, our findings suggest that ovarian androgen production not only declines with menopause but also with older age [16].

The present findings, showing a significant reduction in ovarian volume in patients with MetS, are in agreement with results showing delayed menarche and irregular menstruation in patients with type 1 diabetes [17] and significant reductions in ovarian volume and AFC in patients with type 2 diabetes aged 20–29 years [1], compared with age-matched controls. In contrast, ovarian volumes in subjects aged 20–39 and 40– 49 years were similar in those with MetS and healthy controls. Type I diabetes patients have also been shown to have a high risk of premature menopause [18].

A previous study of Turkish women aged 16 to 40 years found that ovarian volumes were significantly lower among patients with than without polycystic ovarian syndrome (12.5 vs. 5.4 cm^3^) [19]. Another study involving 62 infertile and 53 fertile women aged 35 to 45 years found that mean AFC counts and FSH levels were similar, while ovarian volume was significantly lower in infertile than in fertile women (1.8 vs. 6.1 cm^3^) [20].

Since ovarian volume is a parameter used in traditional in vitro fertilization methods [21], age and ovarian volume were found to be negatively correlated with subject age [22]. Ovarian volume and AFC both decreased with age in our study population. A Brazilian study reported that mean ovarian volume was 7.1 cm^3^ during the perimenopausal period, decreasing 0.2cm^3^ each year [23]. A study in China of 31 healthy volunteers aged 22 to 42 years, found that AFC declined at a rate of 0.95 follicles/year or 60% [24], while another study of infertile Chinese women showed that AFC declined 0.35 follicles/year or 3.8% [25,26].

Various mechanisms may be responsible for the reduction in ovarian reserve among diabetic women. Chronic complications and prolonged hyperglycemia have been reported to negatively affect ovarian reserve, with regular blood glucose control suggested to improve fertility and menstruation anomalies [17].

Polycystic ovarian reserve and diabetes prevalence was reported to increase with increasing obesity, resulting in reductions in oocyte quality [27]. Diabetes related increases in menstruation anomalies and the risk of premature menopause were shown to be associated with increased cardiovascular risks [28]. In agreement with the significantly higher testosterone concentrations we observed in MetS patients than in controls, a Chinese study of 719 women with polycystic ovary syndrome and 685 healthy volunteers found that patients with polycystic ovary syndrome and obesity had higher serum testosterone and fasting insulin levels, lower LH levels, and enlarged ovarian follicles compared with control subjects [24]. In many studies on patients undergoing invitro fertilization procedures, increased BMI was found to have a negative impact on ovarian reserve [29]. Moreover, inhibin B levels and AFC were significantly lower in overweight patients [29]. Indeed, we found that ovarian volume was greater in patients with BMI <30 kg/m^2^ than in subjects with higher BMI.

Although the impact of obesity on the female reproductive system remains unclear, obesity is considered a risk factor for poorer overall health, which, in turn, has negative effects on reproduction, menstrual function, ovulation, and GnRH regulation [30]. Obesity is also an independent risk factor for PCOS and plays important roles in the clinical, metabolic, and biochemical changes that occur throughout PCOS [31]. The finding of significantly higher BMI and waist circumference among patients with MetS and type II diabetes [1,32] accords with thehigh prevalence of infertility and low chance of pregnancy of such patients, even using assisted reproductive technologies [33].

In conclusion, we found that ovarian reserve was lower in patients with MetS than in healthy control subjects, especially in those aged 20–29 years. We also observed a positive correlation between ovarian volume and AFC and negative correlations of both with age. Since a greater ovarian reserve has been associated with a healthier cardiometabolic risk factor profile [12], future larger scale studies are needed to clarify the role of obesity in the association between MetS and ovarian reserve, along with other likely determinants of this interaction.

## Competing interests

The authors declare that they have no competing interests.

## Authors’ contributions

FB collected patients, and wrote the article, NC made USG to patients, CAU wrote article and made statistics, drafted the manuscript, OKU collected patients, BU collected patients, designed study. All authors read and approved the final manuscript.

## References

[B1] Isik S Ozcan HN Ozuguz U Tutuncu YA Berker D Alimli AG Akbaba G Karademir MA Guler S Evaluation of ovarian reserve based on hormonal parameters, ovarian volume, and antral follicle count in women with type 2 diabetes mellitus J Clin Endocrinol Metab 2012 97 261 269 10.1210/jc.2011-1923 22031524

[B2] Bulun SE Adashi EY Larson PR, Kronenberg HM, Melmed S, Polonsky KS The physiology and pathology of the female reproduction axis Williams textbook of endocrinology 2003 10 Philadelphia, PA: Elsevier Science 587 664

[B3] Templeton A Morris JK Parslow W Factors that affect outcome of in vitro fertilisation treatment Lancet 1996 348 1402 1406 10.1016/S0140-6736(96)05291-9 8937279

[B4] Lass A Skull J McVeigh E Margara R Winston RM Measurement of ovarian volume by transvaginal sonography before ovulation induction with human menopausal gonadotrophin for in vitro fertilization can predict poor response Hum Reprod 1997 12 294 297 10.1093/humrep/12.2.294 9070714

[B5] Hendriks DJ Kwee J Mol BW te Velde ER Broekmans FJ Ultrasonography as a tool for the prediction of outcome in IVF patients: a comparative meta-analysis of ovarian volume and antral follicle count Fertil Steril 2007 87 764 775 10.1016/j.fertnstert.2006.11.006 17239869

[B6] Hendriks DJ Mol BW Bancsi LF TeVelde ER Broekmans FJ Antral follicle count in the prediction of poor ovarian response and pregnancy after in vitro fertilization: a meta-analysis and comparison with basal follicle stimulating hormone level Fertil Steril 2005 83 291 301 10.1016/j.fertnstert.2004.10.011 15705365

[B7] Broekmans FJ Kwee J Hendriks DJ Mol BW Lambalk CB A systematic review of tests predicting ovarian reserve and IVF outcome Hum Reprod Update 2006 12 685 718 10.1093/humupd/dml034 16891297

[B8] Reaven GM Banting lecture. Role of insulin resistance in human disease Diabetes 1988 37 1595 1607 10.2337/diab.37.12.1595 3056758

[B9] Hu G Qiao Q Tuomilehto J The metabolic syndrome and cardiovascular risk Curr Diabetes Rev 2005 1 137 143 10.2174/1573399054022820 18220589

[B10] Masheshwari A Stofberg L Bhattacharya S Effect of overweight and obesity on assisted reproductive technology – a systematic review Hum Reprod Update 2007 13 433 444 10.1093/humupd/dmm017 17584821

[B11] Jungheim ES Travieso JL Carson KR Moley KH Obesity and reproductive function Obstet Gynecol Clin North Am 2012 39 479 493 10.1016/j.ogc.2012.09.002 23182555 PMC3520133

[B12] Bleil ME Gregorich SE McConnell D Rosen MP Cedars MI Does accelerated reproductive aging underlie premenopausal risk for cardiovascular disease? Menopause 2013 20 1139 1146 10.1097/GME.0b013e31828950fa 23591257 PMC4123819

[B13] Expert Panel on Detection, Evaluation, and Treatment of High Blood Cholesterol in Adults Executive Summary of the Third Report of the National Cholesterol Education Program (NCEP) Expert Panel on Detection, Evaluation, and Treatment of High Blood Cholesterol in Adults (Adult Treatment Panel III) J Am Med Assoc 2001 285 2486 2497 10.1001/jama.285.19.2486 11368702

[B14] Matthews DR Hosker JP Rudenski AS Naylor BA Treacher DF Turner RC Homeostasis model assessment: insulin resistance and beta-cell function from fasting plasma glucose and insulin concentrations in man Diabetologia 1985 28 412 419 10.1007/BF00280883 3899825

[B15] Gokcel A Ozsahin AK Sezgin N Karakose H Ertorer ME Akbaba M Baklaci N Sengul A Guvener N High prevalence of diabetes in Adana, a southern province of Turkey Diabetes Care 2003 26 3031 3034 10.2337/diacare.26.11.3031 14578235

[B16] Shifren JL Schiff I The aging ovary J Womens Health Gend Based Med 2000 1 S3 S7 10695867 10.1089/152460900318795

[B17] Yeshaya A Orvieto R Dicker D Karp M Ben-Rafael Z Menstrual characteristics of women suffering from insulin-dependent diabetes mellitus Int J Fertil Menopausal Stud 1995 40 269 273 8556032

[B18] Dorman JS Steenkiste AR Foley TP Strotmeyer ES Burke JP Kuller LH Familial Autoimmune and Diabetes (FAD) Study. Menopause in type 1 diabetic women: is it premature? Diabetes 2001 50 1857 1862 10.2337/diabetes.50.8.1857 11473049

[B19] Köşüş N Köşüş A Turhan NÖ Kamalak Z Do threshold values of ovarian volume and follicle number for diagnosing polycystic ovarian syndrome in Turkish women differ from western countries? Eur J Obstet Gynecol Reprod Biol 2011 154 177 181 10.1016/j.ejogrb.2010.10.007 21051133

[B20] Erdem M Erdem A Biberoglu K Arslan M Age-related changes in ovarian volume, antral follicle counts and basal follicle stimulating hormone levels: comparison between fertile and infertile women Gynecol Endocrinol 2003 17 199 205 12857427 10.1080/gye.17.3.199.205

[B21] Syrop CH Willhoite A Van Voorhis BJ Ovarian volume: a novel outcome predictor for assisted reproduction Fertil Steril 1995 64 1167 1171 7589671 10.1016/s0015-0282(16)57979-5

[B22] Kupesic S Kurjak A Bjelos D Vujisic S Three-dimensional ultrasonographic ovarian measurements and in vitro fertilization outcome are related to age Fertil Steril 2003 79 190 197 10.1016/S0015-0282(02)04567-3 12524087

[B23] Oppermann K Fuchs SC Spritzer PM Ovarian volume in pre- and perimenopausal women: a population-based study Menopause 2003 10 209 213 10.1097/00042192-200310030-00006 12792291

[B24] Malhotra N Bahadur A Singh N Kalaivani M Mittal S Does obesity compromise ovarian reserve markers? A clinician’s perspective Arch Gynecol Obstet 2013 287 161 166 10.1007/s00404-012-2528-7 22930149

[B25] Ng EH Yeung WS Fong DY Ho PC Effects of age on hormonal and ultrasound markers of ovarian reserve in Chinese women with proven fertility Hum Reprod 2003 18 2169 2174 10.1093/humrep/deg404 14507840

[B26] Chang MY Chiang CH Chiu TH Hsieh TT Soong YK The antral follicle count predicts the outcome of pregnancy in a controlled ovarian Hyperstimulation/intrauterine insemination program J Assist Reprod Genet 1998 15 12 17 10.1023/A:1022518103368 9493060 PMC3468200

[B27] Metwally M Cutting R Tipton A Skull J Ledger WL Li TC Effect of increased body mass index on oocyte and embryo quality in IVF patients Reprod Biomed Online 2007 15 532 538 10.1016/S1472-6483(10)60385-9 18044034

[B28] Van der Schouw YT van der Graaf Y Steyerberg EW Eijkemans JC Banga JD Age at menopause as a risk factor for cardiovascular mortality Lancet 1996 347 714 718 10.1016/S0140-6736(96)90075-6 8602000

[B29] Zhang HY Guo CX Zhu FF Qu PP Lin WJ Xiong J Clinical characteristics, metabolic features, and phenotype of Chinese women with polycystic ovary syndrome: a large-scale case–control study Arch Gynecol Obstet 2013 287 525 531 10.1007/s00404-012-2568-z 23108387

[B30] Brewer CJ Balen AH The adverse effects of obesity on conception and implantation Reproduction 2010 140 47 364 10.1530/REP-09-0568 20395425

[B31] Wojciechowski P Lipowska A Rys P Ewens KG Franks S Tan S Lerchbaum E Vcelak J Attaoua R Straczkowski M Azziz R Barber TM Hinney A Obermayer-Pietsch B Lukasova P Bendlova B Grigorescu F Kowalska I Goodarzi MO Strauss JF 3rd McCarthy MI Malecki MT GIANT Consortium Impact of FTO genotypes on BMI and weight in polycystic ovary syndrome: a systematic review and meta-analysis Diabetologia 2012 55 636 2645 10.1007/s00125-011-2404-1 22801903 PMC3433670

[B32] Ding EL Song Y Malik VS Liu S Sex differences of endogenous sex hormones and risk of type 2 diabetes: a systematic review and meta-analysis JAMA 2006 295 1288 1299 10.1001/jama.295.11.1288 16537739

[B33] Wang JX Davies M Norman RJ Body mass and probability of pregnancy during assisted reproduction treatment: retrospective study BMJ 2000 321 1320 1321 10.1136/bmj.321.7272.1320 11090515 PMC27536

